# Study of the improved Sf9 transient gene expression process

**DOI:** 10.1186/1753-6561-7-S6-P19

**Published:** 2013-12-04

**Authors:** Xiao Shen, David L Hacker, Lucia Baldi, Florian M Wurm

**Affiliations:** 1 Laboratory of Cellular Biotechnology, Faculty of Life Sciences, Ecole Polytechnique Fédérale de Lausanne, CH-1015 Lausanne, Switzerland

## Introduction

Insect cells have been widely used for the production of recombinant proteins using recombinant baculovirus for gene delivery [1]. To simplify protein production in insect cells, we have previously described a method, based on transient gene expression (TGE) with cultures of suspension-adapted Sf9 cells using polyethylenimine (PEI) for DNA delivery [2]. Expression of GFP has been realized at high efficiency and a tumor necrosis factor receptor-Fc fusion protein (TNFR-Fc) was produced at a level of 40 mg/L. However, the efficiency of the insect cells TGE system has not been studied and further optimization may improve protein titers. Here, we studied the efficiency of PEI for plasmid delivery in Sf9 cells.

## Methods

### Cell culture

Sf-9 cells were maintained in suspension in TubeSpin^® ^bioreactor 600 at 28°C [3].

### Sf-9 cells Transfection

Sf9 cells were transfected as described before [2] using 25 kDa polyethylenimine PEI (Polysciences, Warrington, PA) and an expression vector for GFP or TNFR-Fc. GFP-specific fluorescence was measured 48 h post-transfection using the GUAVA EasyCyteTM flow cytometer (Millipore, Billerica MA, USA). TNFR-Fc was measured by sandwich ELISA [4].

### Estimation of plasmid copy number

Total DNA was isolated using DNeasy Blood & Tissue Kit (Qiagen AG, Hombrechtikon, Switzerland) according to the manufacturer's protocol. PCR was executed using the Absolute qPCR SYBR Green ROX reaction mix (Axon Lab AG, Baden-Dättwil, Switzerland) with total cellular DNA as template. The PCR was performed using LightCycler^® ^480 real-time PCR system (Roche Applied Science, Basel, Switzerland). The plasmid copy number was estimated from the standard curve according to the threshold cycle (Ct) of each sample [4].

### Cell cycle analysis

Cells at different times post-transfection were centrifuged and washed with PBS before fixation in 70% ethanol. Fixed cells were washed with PBS and then stained with Guava Cell Cycle Reagent and analyzed by the GUAVA EasyCyteTM flow cytometer. Cells treated with nocodazole (50 ng/mL, 16 h) and mimosine (1 mM, 24 h) were used as references for determining the positions of the G1 and G2/M phases [5].

## Results

### Plasmid delivery efficiency in Sf9 cells

**Figure 1:**
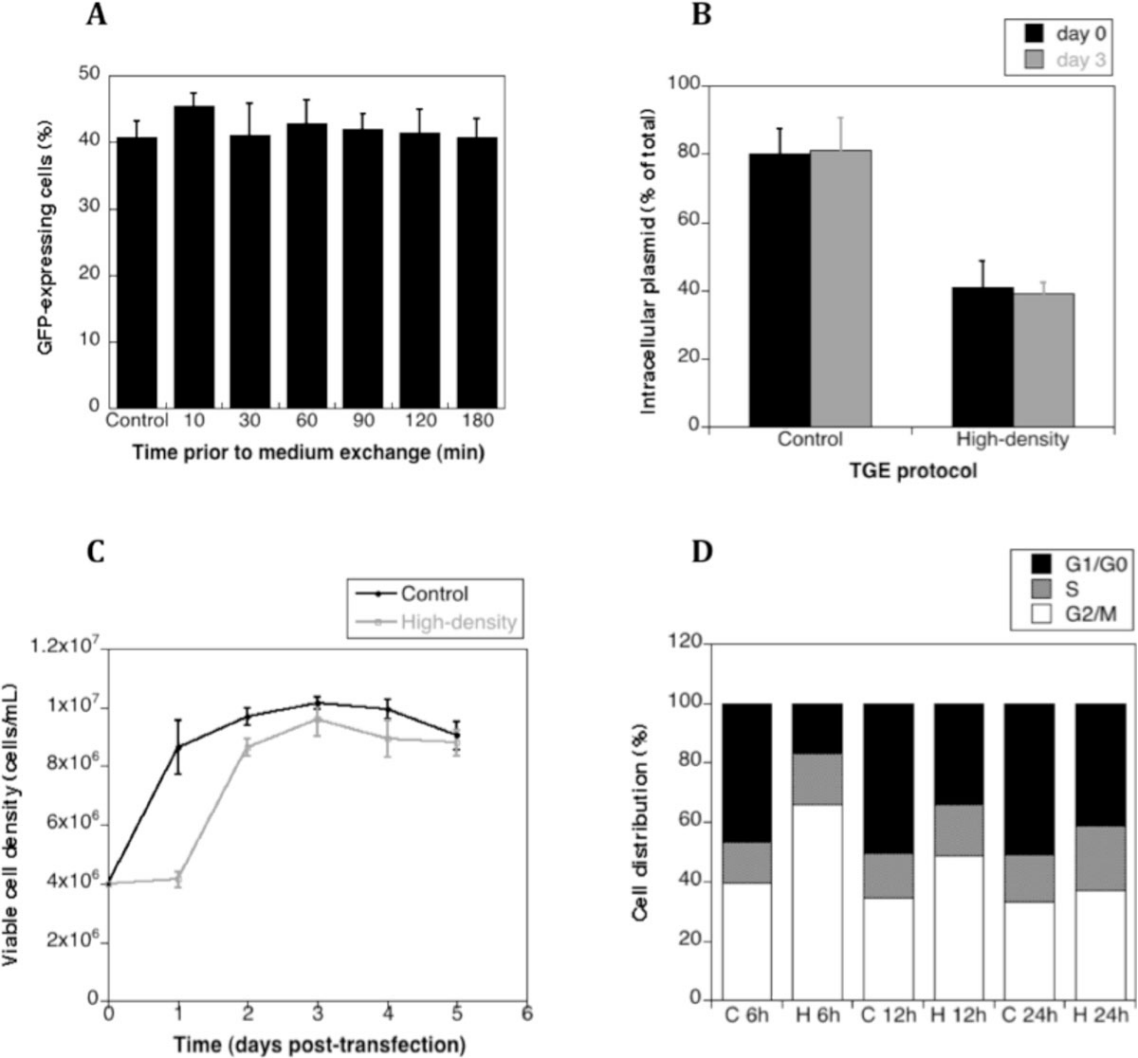
**Study of the Sf9 TGE process**. (A) Sf9 cells were transfected with EGFP-coding plasmid DNA and PEI at a starting cell density of 4 × 10^6 ^cells per ml. Media of the transfected culture were exchanged at 10, 30, 60, 90, 120, 180 minutes post-transfection. EGFP-positive cells were measured on day 2. (B) Average intracellular plasmid copy number on day of transfection and day 3 post-transfection of cultures transfected using control protocol and high-density TGE protocol were analyzed by quantitative PCR. (C) Cell growth of Sf9 cells transfected using the two different protocols were compared. Cell cycle distribution during the first 24 hours post-transfection of those two TGE culture were analyzed (D). C: control transfection at 4 × 10^6 ^cells/mL; H: high-density Sf9 transfection; h: hours.

To measure the time course of plasmid DNA delivery, cells were transfected with a GFP expression vector. At different times post-transfection, a complete medium exchange was performed. The percentage of GFP-positive cells was determined for all cultures including a control for which a medium exchange was not performed. All cultures exhibited similar levels of GFP-positive cells meaning that DNA uptake into cells occurred within 10 min of DNA addition (Figure 1A).

To measure the amount of DNA uptake, Sf9 cells were transfected in two different ways with a TNFR-Fc expression vector and the amount of intracellular plasmid was measured by quantitative PCR. On the day of transfection more than 80% of the plasmid DNA was present within cells with the control transfection while 40% of the DNA was present within cells following a high-density transfection (Figure 1B). It has been reported that improved plasmid delivery can result in an increase in specific and volumetric productivity for HEK 293 cells transfected at high-density [6]. However, in our high-density protocol, plasmid delivery was diminished in comparison to the control (Figure 1B).

### Plasmid delivery was not improved, but cell growth was inhibited in an optimized TGE process

Improvement in TGE yields from Chinese hamster ovary cells was achieved by reducing the cell growth rate [5,7]. When the cell growth curve of the optimal TGE process with Sf9 cells was compared with that of the control protocol, we observed a significant decrease of viable cell number, within 24 h post-transfection (Figure 1C). This suggested a deregulation in the cell cycle in the initial phase of transfection. The cell cycle distribution was analyzed and an increase of the percentage of cells in the G2/M phase was observed for the high-density protocol early after transfection (Figure 1D). However, the growth inhibition was attenuated by 24 h post-transfection (Figure 1D). Nevertheless, the temporary cell growth inhibition contributed to yield improvement in our optimal protocol.

## Conclusion

A previously described method for the transient transfection of Sf9 cells was improved. The increase in recombinant protein yield was not due to an increased plasmid delivery after transfection. However, high-density transfection resulted in a significant percentage of cells being blocked in the G2/M phase of the cell cycle for the first 24 h post-transfection.
